# Unveiling the Origin of pH-Dependent Catalytic Performance of Bi_2_O_3_ Nanostructure for Electrochemical CO_2_ Reduction

**DOI:** 10.1021/acs.jpclett.5c00103

**Published:** 2025-04-07

**Authors:** Nicolò B. D. Monti, Tengfei Chen, Lan Huang, Jun Wang, Marco Fontana, Candido F. Pirri, Wenbo Ju, Juqin Zeng

**Affiliations:** † Department of Applied Science and Technology (DISAT), Politecnico di Torino, Corso Duca degli Abruzzi 24, Turin 10129, Italy; ‡ School of Physics and Optoelectronics, South China University of Technology, Wushan Road 381, Tianhe District, Guangzhou 510641, China; § Istituto Italiano di Tecnologia−IIT, Centre for Sustainable Future Technologies (CSFT), Via Livorno 60, Turin 10144, Italy

## Abstract

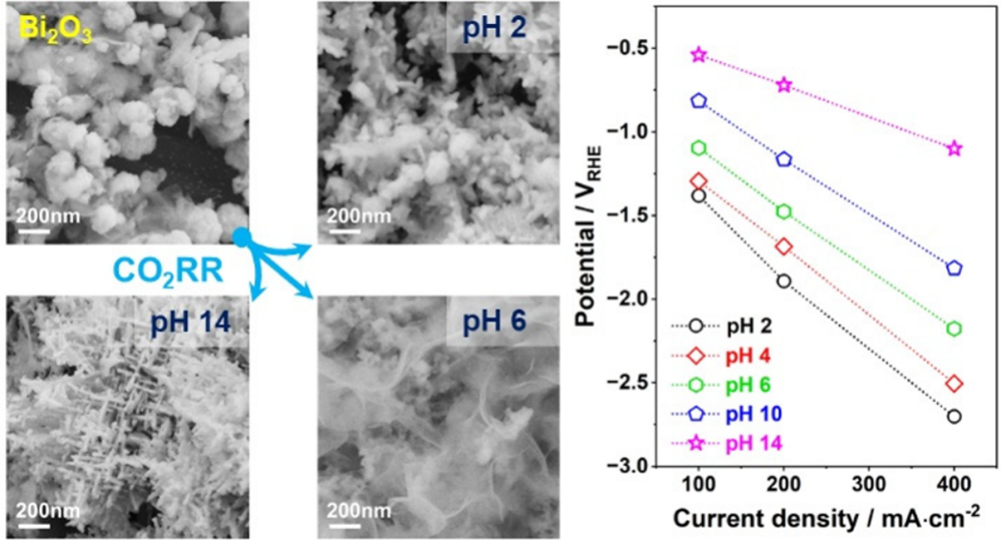

Over the past decade, the electrochemical conversion of CO_2_ into valuable chemicals and fuels has garnered increasing interest as a promising pathway toward a carbon-neutral circular economy. This study investigates Bi_2_O_3_ gas diffusion electrodes (Bi-GDEs) for the conversion of CO_2_ to formic acid/formate (HCOOH/HCOO^–^), which demonstrate excellent selectivity at high current densities. The catalyst is synthesized through a one-pot microwave-assisted process that is rapid, energy-efficient, and scalable, utilizing the green solvent ethylene glycol. The resulting Bi_2_O_3_ nanostructure achieves near-unit selectivity for CO_2_ conversion, with a faradaic efficiency exceeding 95% for HCOOH/HCOO^–^ formation across a wide pH range. The catalytic activity is strongly pH-dependent, with an increase in the pH reducing the overpotential at a given current density. To elucidate the origin of this pH-dependent activity, *operando* Raman spectroscopy, *post-mortem* scanning electron microscopy (SEM), and electrical double layer characterization were performed. *Operando* Raman results reveal that Bi_2_O_3_ undergoes reduction more readily in highly acidic or basic electrolytes, whereas its reduction is inhibited near neutral pH. However, at highly negative potentials relevant to CO_2_RR, cationic Bi species fully convert to metallic Bi. Despite structural variations at different electrolyte pH values, metallic Bi remains the active phase, explaining the high selectivity of Bi-GDEs across a broad pH range. *Post-mortem* SEM images highlight the influence of electrolyte pH on morphological evolution under CO_2_RR conditions. At the highest pH of 14, a hierarchical dendritic structure emerges, showing an increase of 100% in double layer capacitance, which evidences the significant enhancement in the electrochemical active surface area and consequently the CO_2_RR activity.

Over the past decade, interest in the electrochemical carbon dioxide reduction reaction (CO_2_RR) has grown due to its potential to close the carbon cycle by utilizing renewable electricity to produce valuable fuels and chemicals, including carbon monoxide (CO), formic acid/formate (HCOOH/HCOO^–^), ethylene (C_2_H_4_), and ethanol (C_2_H_5_OH).^1−5^ Among these, HCOOH stands out for its economic viability and energy efficiency.^6−8^ It has various commercial applications and holds great promise for hydrogen storage and transportation.^9−11^

Despite its promise, CO_2_RR faces persistent challenges that hinder its progress, particularly its pH dependence.^12^ The reaction is typically conducted in a neutral or basic electrolyte to suppress the competing hydrogen evolution reaction (HER).^13^ Under these conditions, the hydroxide ions (OH^–^) generated near the cathode promote the formation of bicarbonate (HCO_3_^–^) or carbonate (CO_3_^2–^), leading to CO_2_ depletion, salt deposition, and energy penalties associated with regenerating CO_2_ from HCO_3_^–^/CO_3_^2–^. To mitigate these limitations, conducting CO_2_RR in acidic media has emerged as an effective strategy. However, a major challenge is the competing HER, which tends to dominate under acidic conditions.^14^ Consequently, there is a critical need for catalysts with high activity and selectivity for CO_2_RR in acidic environments. Recent efforts have successfully developed bismuth- (Bi-) based materials for the selective conversion of CO_2_ to HCOOH under acidic conditions,^15,16^ offering significant promise for HCOOH/HCOO^–^ production across a broad pH range.^4,17,18^

Another critical challenge in CO_2_RR is the restructuring of catalysts under reaction conditions,^19−21^ which complicates the identification of performance descriptors and impedes further optimization. Recent studies have revealed a clear correlation between the structure and the activity of Bi-based catalysts in neutral electrolyte.^22^ However, the restructuring behavior of Bi-based materials under both acidic and alkaline conditions remains undocumented, and no prior studies have systematically compared the same catalyst across different pH environments.

In this work, we synthesized a Bi_2_O_3_ catalyst using a one-pot microwave-assisted method, employing bismuth(III) nitrate (Bi(NO_3_)_3_) as the precursor and ethylene glycol as the solvent. Detailed synthesis information is provided in the Supporting Information. Field emission scanning electron microscopy (FESEM) and transmission electron microscopy (TEM) reveal that the Bi_2_O_3_ catalyst is predominantly composed of submicrometric spherical particles, with a small fraction of nanosheets (Figures 1a and 1b). Selected area electron diffraction (SAED) and rotationally averaged electron diffraction (Figure 1c and 1d) confirm a tetragonal β-Bi_2_O_3_ phase, with no other crystalline phases detected. X-ray diffraction (XRD) further supports this phase, indexed to the P-421c space group^23^ (Figure 1e). Raman spectroscopy identifies characteristic vibrational modes of Bi_2_O_3_. Bands below 200 cm^–1^ are attributed to the vibrations of Bi^3+^ (91 cm^–1^) and binuclear Bi–O entities (125 cm^–1^) within the oxide matrix (Figure 1f).^24^ The Bi–O stretching and Bi–O–Bi bending modes appear at 315 and 467 cm^–1^, respectively.^24^

**Figure 1:**
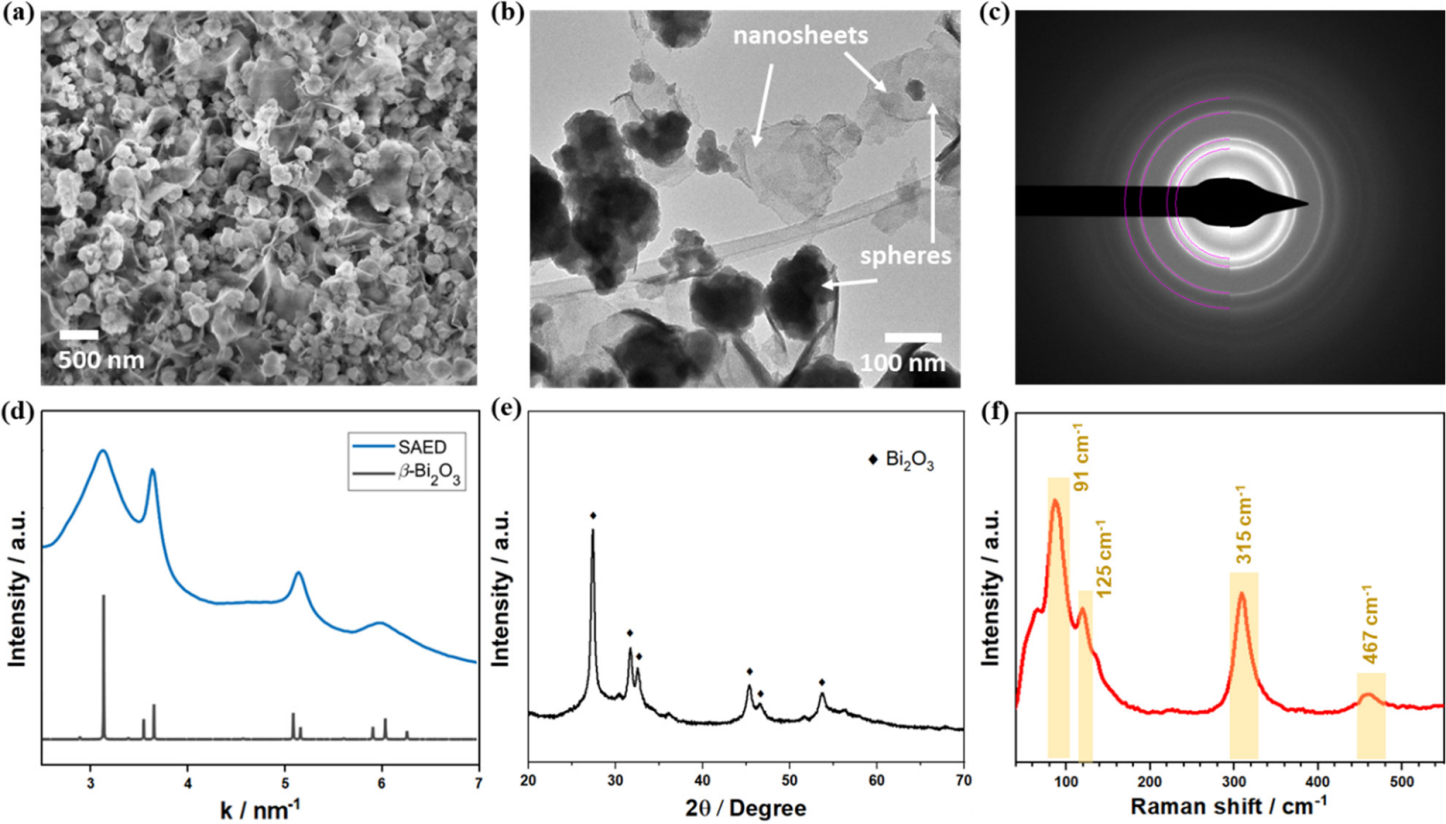
(a) FESEM and (b) bright-field TEM images of the Bi_2_O_3_ catalyst; (c) representative SAED pattern; (d) rotationally averaged electron diffraction profile with reference contributions from the β-Bi_2_O_3_ phase; (e) XRD pattern and (f) Raman spectrum of the Bi_2_O_3_ catalyst.

The Bi_2_O_3_ powder was coated on teflonized carbon paper to fabricate the GDE, as described in the Supporting Information. Bi-GDEs were tested in a customized three-compartment three-electrode flow cell with various electrolytes (Figure S1a). An anion exchange membrane separated the anolyte and catholyte chambers, while the GDE divided the catholyte and CO_2_ gas chambers, with the catalyst layer facing the catholyte. CO_2_ entered from the backside of the GDE, reached the catalyst layer, and underwent electrochemical reduction. Basic electrolytes with different pH values were prepared by neutralizing a 1.0 M potassium hydroxide (KOH) solution with concentrated sulfuric acid (H_2_SO_4_), while acid electrolytes were prepared by adding concentrated H_2_SO_4_ to a 0.5 M potassium sulfate (K_2_SO_4_) solution. The K^+^ concentration remained approximately 1.0 M due to the minimal volume of H_2_SO_4_ added. During testing, the catholyte flowed through the chamber in a single-pass mode.

Figures 2a to 2e show the Faraday efficiencies of CO_2_RR catalyzed by Bi_2_O_3_ powder in electrolytes of varying pH. At pH 2, the primary liquid product is HCOOH (Figure 2a). The HCOOH selectivity increases from 68% at 100 mA·cm^–2^ to 95% at 400 mA·cm^–2^, while CO selectivity remains constant at 2 ∼ 3%. Bi-GDEs achieve a total Faradaic efficiency exceeding 97% for CO_2_RR, with HCOOH/HCOO^–^ as the primary product, across a broad pH range (4 to 14) and current densities (100 to 400 mA·cm^–2^), demonstrating the highly reliable performance of the Bi_2_O_3_ catalyst.

**Figure 2:**
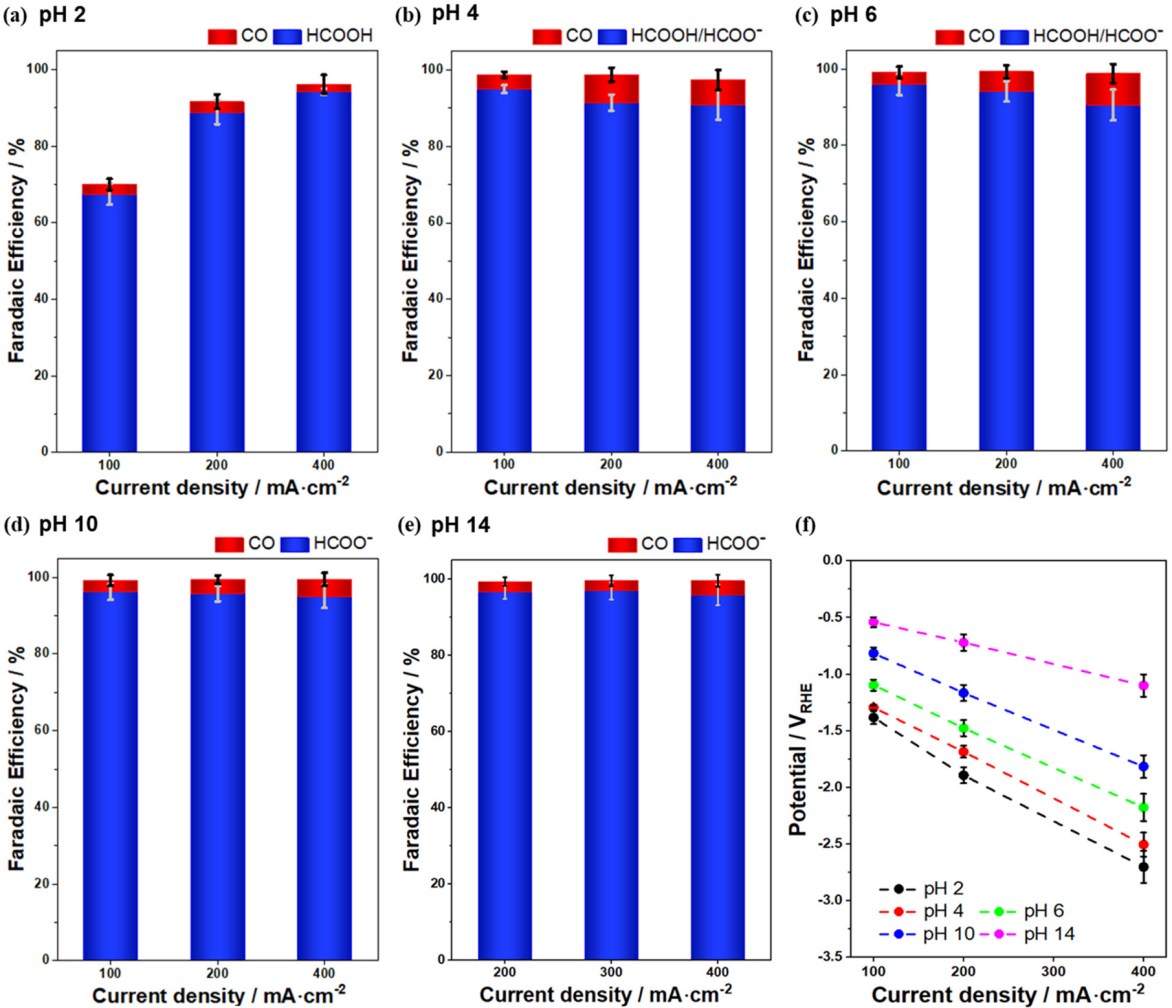
Faradaic efficiencies of CO_2_RR on Bi-GDE at different current densities in various electrolytes: (a) pH 2, (b) pH 4, (c) pH 6, (d) pH 10 and (e) pH 14; (f) Electrode potentials recorded at different current densities in various electrolytes.

Figure 2f illustrates the electrode potentials on Bi-GDEs at different current densities in various electrolytes. As the current density increases, the potential becomes more negative, indicating a higher electrochemical driving force for faster reaction rates. At each current density, the required potential varies with pH, with lower-pH electrolytes necessitating more negative potentials to achieve the same reaction rate as higher-pH electrolytes. This suggests that the Bi-GDE is more active under basic conditions, consistent with the linear sweep voltammetry results shown in Figure S2. Bi-GDE activity increases slightly from pH 2 to 4, followed by a significant enhancement at higher pH.

Bi-GDEs were also tested in the flow cell with a two-electrode configuration (Figure S1b). A constant current density of 400 mA·cm^–2^ was applied, and the cell voltage was recorded. As shown in Figure S3, the voltage remains steady throughout each test, with consistently high selectivity for HCOOH/HCOO^–^. At low pH, the cell voltage ranges from 3.6 to 3.8 V, whereas it drops significantly to approximately 2.7 V at pH 14. These results align with those from the three-electrode measurements, confirming that Bi_2_O_3_ exhibits higher activity for CO_2_RR to HCOOH/HCOO^–^ at elevated pH levels. It is also worth noting that the Bi-GDE demonstrates good stability over 8 hours at a high current density of 400 mA·cm^–2^, with H_2_ selectivity remaining below 10% in the pH 2 electrolyte. Increasing the pH reduces durability, as higher pH facilitates more rapid CO^_3_2–^/HCO_3_^–^ salt deposition, which clogs the electrode and compromises the overall longevity of the electrolysis.

Since the Bi-GDEs exhibit varying performance across different pH levels, *operando* electrochemical Raman spectroscopy was conducted using a three-electrode setup to investigate the compositional evolution as a function of applied potential in different electrolytes. The Bi_2_O_3_ catalyst was coated onto carbon paper, with the catalyst layer exposed to CO_2_ and the laser light path. Details of the Raman spectroscopy measurements are provided in the Supporting Information.

Figure 3 shows a potential-dependent series of Raman spectra for Bi-GDEs exposed to electrolytes with different pH values. At pH 2, the spectra of the supported catalysts at high potentials are similar to those of the as-prepared Bi_2_O_3_ powder. At lower potentials, the bands at 71 and 98 cm^–1^ appear, corresponding to metallic Bi,^18^ as verified by the Raman spectrum of metallic Bi powder (Figure S4). The potential-dependent Raman intensity (Figure 4a) reveals that Bi_2_O_3_ dominates the surface at potentials above 0.21 V, while metallic Bi becomes dominant at lower potentials. The Raman band at 980 cm^–1^ corresponds to SO_4_^2–^ in the solution, and its intensity remains unchanged with the applied potential (Figure S5).

**Figure 3:**
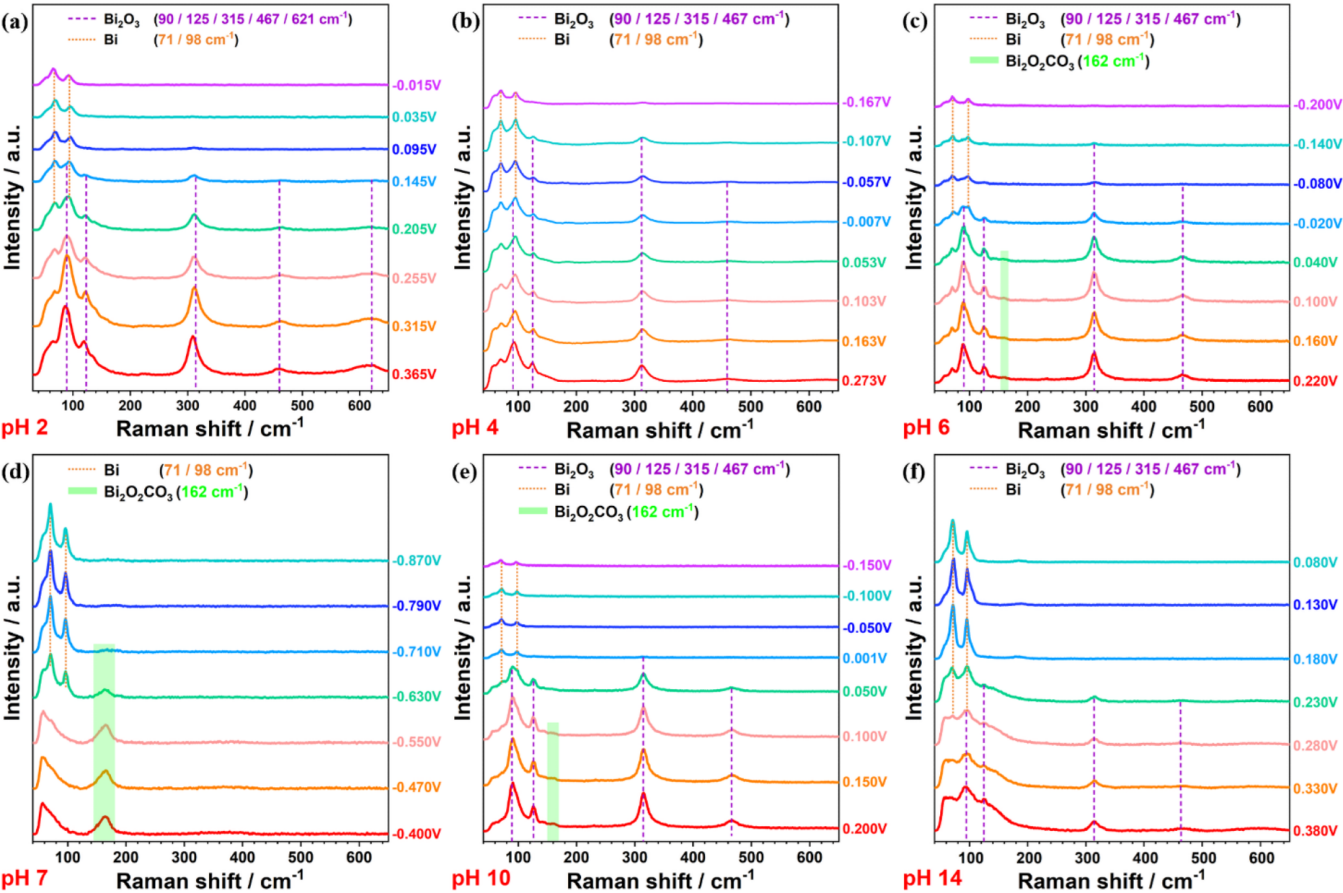
*Operando* electrochemical Raman spectra of Bi-GDEs in different electrolytes: (a) pH 2, (b) pH 4, (c) pH 6, (d) pH 7, (e) pH 10, and (f) pH 14.

**Figure 4:**
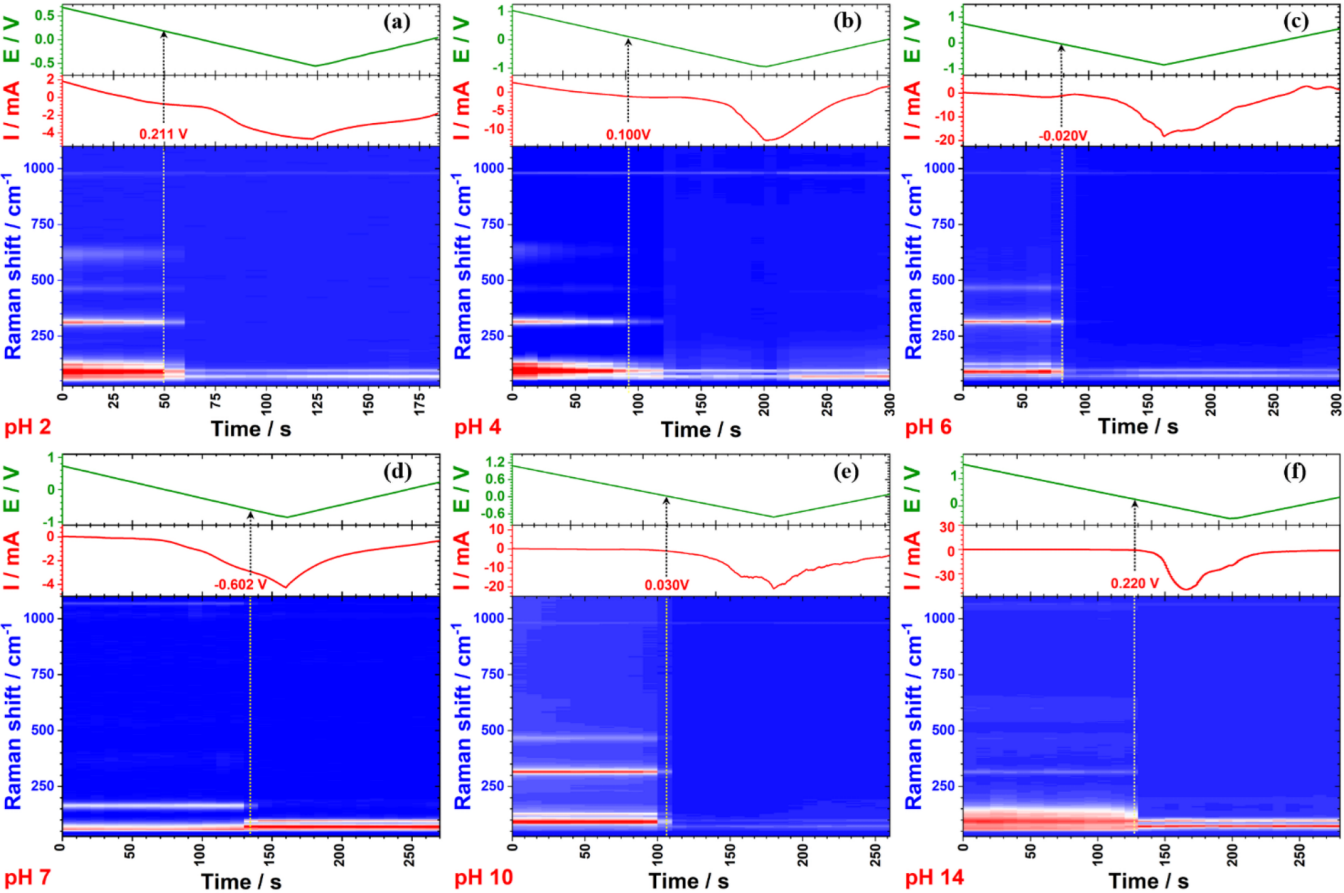
Raman intensity of Bi-GDEs in different electrolytes: (a) pH 2, (b) pH 4, (c) pH 6, (d) pH 7, (e) pH 10 and (f) pH 14.

Bi_2_O_3_ dominates the surface composition at high potentials and reduces to metallic Bi under reductive conditions across various pH values, as shown in Figure 3 (except 3d) and Figure 4 (except 4d). However, the reduction potential of Bi_2_O_3_ is pH-dependent. As the electrolyte approaches neutral pH, the reduction potential becomes more negative. The potential at which the Raman intensity of Bi_2_O_3_ decreases by 50% is considered the threshold for phase transition. When adjusting the electrolytes from pH 2 to pH 6, the potential for phase transition shifts from above 0.20 V to approximately–0.02 V. In the pH 10 electrolyte, the phase transition occurs slightly above 0.0 V, similar to what is observed in the pH 6 electrolyte. Additionally, a small amount of subcarbonate species (Bi_2_O_2_CO_3_) is observed on the catalysts in pH 6 and pH 10 electrolytes at high potentials, as indicated by the Raman band at 162 cm^–1^ (Figure 3c and 3e). However, the Bi_2_O_2_CO_3_ overlayer on Bi_2_O_3_ particles is too thin to be effectively characterized by the nonsurface-sensitive techniques such as XRD. In the highly basic electrolyte (pH 14), the phase transition is similar to that in the highly acidic electrolyte (pH 2), occurring at potentials more positive than 0.20 V. The pH 7 electrolyte, which is CO_2_-saturated 1.0 M KHCO_3_, causes Bi_2_O_3_ nanoparticles to spontaneously convert into Bi_2_O_2_CO_3_. The reduction of Bi_2_O_2_CO_3_ to metallic Bi occurs within the potential range from–0.47 to–0.69 V, with the phase transition occurring around–0.60 V, which is more negative than in other electrolytes. The electrochemical reduction of commercial Bi_2_O_2_CO_3_ powder was tested using *operando* Raman spectroscopy during cyclic voltammetry in neutral (pH 7) and alkaline (pH 14) electrolytes (Figure S6). In the pH 7 electrolyte, Bi_2_O_2_CO_3_ is reduced to metallic Bi at approximately–0.52 V, consistent with the results shown in Figure 4d. The transition of Bi_2_O_2_CO_3_ to metallic Bi occurs at around–0.50 V in pH 14 electrolyte, showing minimal dependence on the pH. However, the reduction potential is more negative compared to that of Bi_2_O_3_ in the same solution (Figure 4f). These Raman results indicate that Bi_2_O_3_ is more readily reduced in highly acidic or basic electrolytes, while its reduction becomes more difficult as the pH approaches neutrality. Bi_2_O_2_CO_3_ can serve as a passivation layer, inhibiting the phase transition from Bi_2_O_3_ to Bi.

The dynamic changes in the composition of catalysts during electrochemical reduction were quantitatively analyzed using Raman intensity with Boltzmann combination fitting (BCF). Raman peak intensities were normalized to the highest peak value in each spectrum. The BCF results, derived from the potential-dependent Raman spectra, are shown in Figure S7. At pH 2, the compositional evolution begins at 0.30 V, with a transition state (a mixture of Bi_2_O_3_ and metallic Bi phases) observed between 0.30 and 0.04 V. In contrast, at pH 14, the transition state occurs between 0.30 and 0.16 V. Notably, the transition state spans a broader potential window at pH 2 and 4 compared to higher pH values. The potential window for the reduction of Bi_2_O_3_ is influenced by the chemical environment, with pH playing a crucial role. Bi^3+^, a Lewis acid, tends to form strong bonds with hydroxide (OH^–^) anions, resulting in the formation of BiOH^2+^, Bi(OH)_2_^+^, Bi(OH)_3_, and Bi(OH)_4_^–^ species across the pH range of 1 to 14.^25^ At low pH, the spontaneous dissolution of Bi-based cations competes with the nucleation and growth of metallic Bi. In contrast, in alkaline environments, Bi–Bi bonds form immediately after the electrochemical cleavage of Bi–O bonds, as evidenced by the small overpotential required to complete the transition state. In particular, ionic Bi species are completely converted to metallic Bi at all pH levels under highly reductive conditions, such as those applied during CO_2_RR. Therefore, despite the differences in compositional evolution across various pH values, metallic Bi remains the active phase for CO_2_RR, exhibiting high selectivity for HCOOH/HCOO^–^.

SEM analysis was performed to examine the pH-dependent morphological evolution of Bi-GDEs (Figure 5). After testing in pH 2 and 4 electrolytes, the supported catalyst retains a particulate morphology, although the particles are smaller than their original size. The dissolution of ionic Bi species, followed by diffusion and redeposition under reductive conditions, leads to the formation of smaller particles. In pH 6 and 10 electrolytes, the catalyst develops interconnected nanosheet structures, indicating a significant morphological transformation. Similarly, after testing in 0.5 M KHCO_3_ electrolyte (pH 7), the Bi-GDE surface becomes uniformly covered with vertically oriented nanosheets, likely due to the formation of Bi_2_O_2_CO_3._^2^ In pH 14 electrolyte, the tested catalyst exhibits hierarchical dendritic structures with diameters of 10 nm and lengths of 200 nm. These well-defined dendrites feature smaller branches growing from larger ones, presenting a distinct morphology compared to the particulate and nanosheet structures observed at lower pH levels. This transformation represents a notable structure evolution of Bi-GDEs during CO_2_RR in alkaline media. A sudden burst of nucleation followed by rapid growth of Bi monomers drives the precipitation of ionic Bi species into dendritic metallic Bi rather than isotropic nanospheres. Generally, nanosheet structures with abundant edges and unsaturated sites can enhance electrocatalytic efficiency. Furthermore, as a low-dimensional structure, Bi-based dendrites possess a large surface area and a high density of active sites, resulting in improved performance compared to particulate Bi morphology.^26−28^ The electric double layer capacitance (*C*_*dl*_) of the electrode after electrolysis was evaluated in the electrolytes with different pH values, as shown in Figure S8. The *C*_*dl*_ increases with raising the electrolyte pH. Particularly, the *C*_*dl*_ is doubled in pH 14 with respect to the one in pH 2, evidencing the significant enhancement in electrochemically active surface area (ECSA) at high pH. The enhanced ECSA leads to more active sites and thus improves the activity of the whole electrode. Additionally, dendritic morphology can strengthen the interfacial electric field due to the tip effect, where charge concentration increases in regions with higher curvature on uneven electrodes.^29−32^ As the material sharpens, the electron density rises, generating a morphology-dependent electric field through electrostatic interactions between electrons and cations. This field is typically stronger at sharper regions than on smoother electrode surfaces, significantly altering the local electrochemical environment and modulating CO_2_RR energy profiles, enhancing CO_2_RR activity.

**Figure 5:**
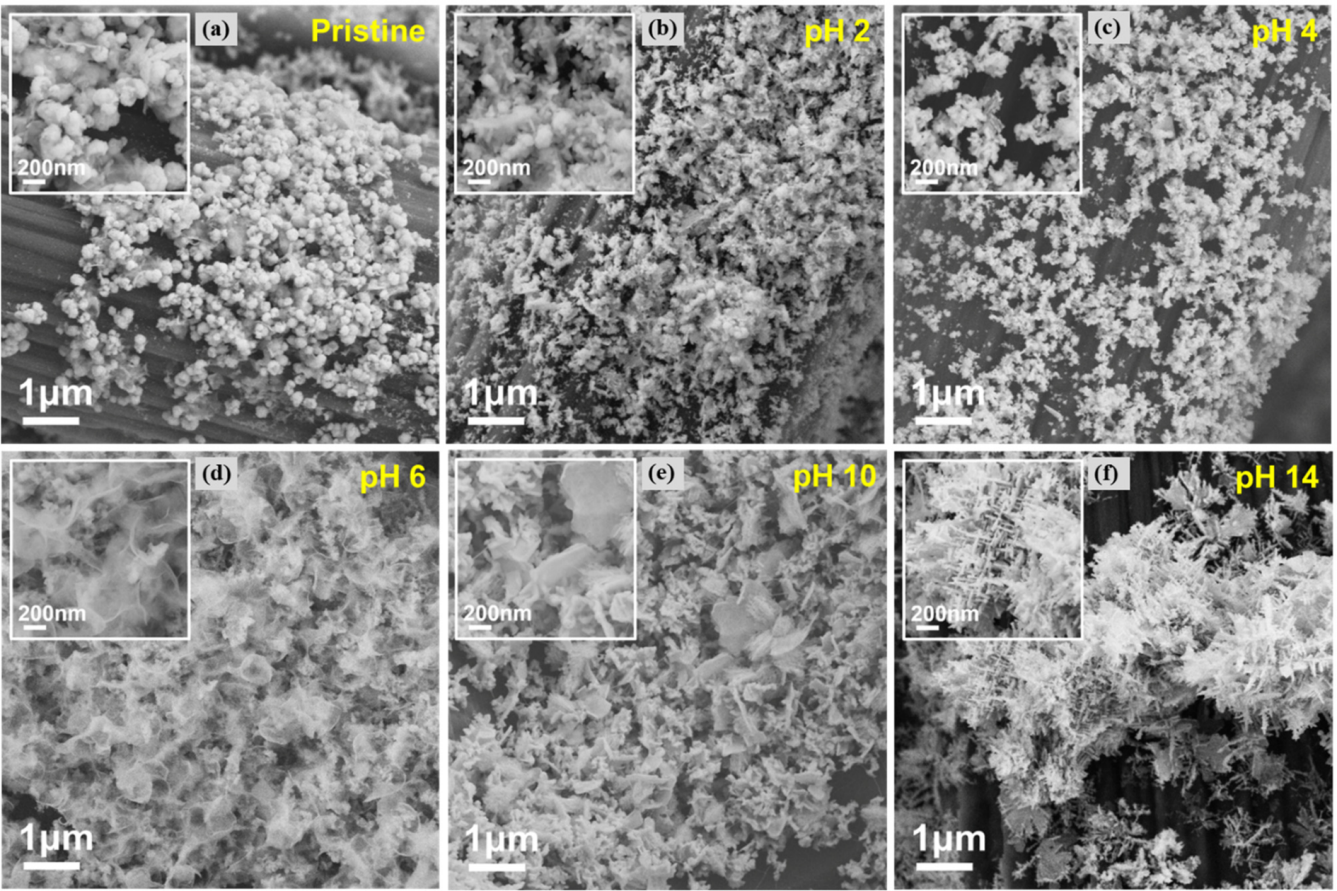
SEM images of Bi-GDEs after tests in different electrolytes: (a) Pristine, (b) pH 2, (c) pH 4, (d) pH 6, (e) pH 10 and (f) pH 14.

In summary, Bi_2_O_3_ nanostructures are prepared for HCOOH/HCOO^–^ production via electrochemical CO_2_RR. Bi-GDEs achieve excellent selectivity (>95%) for target products at industrially relevant conversion rates (200–400 mA·cm^–2^), regardless of electrolyte pH. However, higher pH reduces the overpotential at the same current density, indicating pH-dependent activity. *Operando* Raman spectra confirm that metallic Bi remains the active phase despite structural variations across different pH values, explaining the consistently high selectivity over a broad pH range. The morphology of restructured Bi-GDEs is influenced by electrolyte pH, transitioning from nanospheres under acidic conditions to vertically oriented nanosheets in neutral electrolytes and dendritic structures in strongly alkaline environments. This underscores the critical role of pH in morphological evolution under CO_2_RR conditions. As pH increases, the formation of nanosheets or hierarchical dendrites provides a highly porous structure with an extensive ECSA and abundant active sites, enhancing CO_2_RR activity. In particular, dendritic morphology benefits from an intensified local electric field at structural tips, further promoting CO_2_RR. These insights into the compositional and morphological evolution of catalysts offer valuable guidance for designing next-generation catalysts for this application.

## Data Availability

The data sets generated during and/or analyzed during the current study are available from the corresponding authors upon reasonable request.

## Supporting Information Available

Details on experimental methods (synthesis and physicochemical characterization, electrochemical characterization and *operando* Raman spectroscopy), schemes of the electrochemical cell, linear sweeping voltammetry study on Bi-GDE in electrolytes with different pH values in a flow cell, CO_2_RR on Bi-GDE with a two-electrode mode and Boltzmann combination fitting results of the potential-dependent *operando* Raman spectra on Bi-GDEs in different electrolytes (PDF)
