# Supplementary material for: Unveiling the Origin of pH-Dependent Catalytic Performance of Bi2O3 Nanostructure for Electrochemical CO2 Reduction
Source: J Phys Chem Lett. 2025 Apr 7;16(15):3761–8. doi: 10.1021/acs.jpclett.5c00103 (PMC12010420; doi:10.1021/acs.jpclett.5c00103)
Supplement: Supplementary file 1 — jz5c00103_si_001.pdf [file jz5c00103_si_001.pdf]

Supporting information

Unveiling the Origin of pH-dependent Catalytic Performance of Bi_2O_3 Nanostructure for Electrochemical CO_2 Reduction

Nicolò B. D. Monti,[†,§,∥], Tengfei Chen,[‡,∥] Lan Huang,[†,§] Jun Wang,[‡] Marco Fontana,[†,§] Candido F. Pirri,[†,§] Wenbo Ju,[*,‡] Juqin Zeng[*,†,§]

[†]Department of Applied Science and Technology (DISAT), Politecnico di Torino, Corso Duca degli Abruzzi 24, Turin,10129, Italy

[‡]School of Physics and Optoelectronics, South China University of Technology, Wushan Road 381, Tianhe District, Guangzhou, 510641, China

[§]Istituto Italiano di Tecnologia – IIT, Centre for Sustainable Future Technologies (CSFT), Via Livorno 60, Turin, 10144, Italy

[∥]*N.B.D. Monti and T. Chen contributed equally.*

[*]*Corresponding author:* Juqin Zeng (juqin.zeng@polito.it), Wenbo Ju (wjuphy@scut.edu.cn)

1. Synthesis and physicochemical characterization

1.1. Materials

Bismuth (III) nitrate pentahydrate ($Bi(NO_3)_3{\cdot}5H_2O$, 98 wt.%), ethylene glycol (EG, 99.8 wt.%), acetic acid (CH_3COOH, 99.8 wt.%, anhydrous), Nafion® 117 solution (5 wt.%), isopropanol and potassium hydroxide (KOH, > 85 wt.%) and sulfuric acid (H_2SO_4, 98 wt.%) were purchased from Sigma-Aldrich (Merck). Metallic Bismuth powder (99.5%, 325 mesh) was purchased from Leyan-Chem. Bismuth subcarbonate ($Bi_2O_2CO_3$, AR 90.0%) was supplied by Macklin. All the chemicals were used as received.

1.2. Synthesis process

In a typical synthesis, 900 mg of bismuth nitrate pentahydrate were dissolved in 40 mL of EG, 2 mL water and 2 mL of acetic acid. Then, the precursor solution was transferred into a Teflon vessel (volume 100 mL) that was placed into a microwave oven and connected to pressure and temperature probes (Milestone STARTSynth, Milestone Inc., Shelton, Connecticut). The Teflon vessel was then irradiated for 2 min at controlled conditions (maximum temperature of 220 °C and maximum power of 900 W). After cooling down to ambient temperature, the precipitate was separated by centrifuge and washed twice with water and once with ethanol. The powder sample was finally obtained by vacuum dry at 60 °C overnight.

1.3. Physical and chemical characterization

The morphology study of the as-prepared sample was carried out by means of Field Emission Scanning Electron Microscopy (FESEM) with a Zeiss Supra 40 microscope (Zeiss, Oberkochen, Germany). X-Ray diffraction analysis (XRD) was performed to determine the crystalline phases of the as-prepared and restructured electrodes by using a PANalytical X'Pert Pro instrument (Cu-Kα radiation, 40 kV and 40 mA) equipped with an X'Celerator detector.

Transmission Electron Microscopy (TEM) characterization was performed with a TALOS F200X instrument (Thermo Fisher, Waltham, MA, USA). The analysis of the images was carried out with the Thermo Scientific Velox software while the electron diffraction patterns were analyzed with

Gatan Microscopy Suite. The rotationally-averaged electron diffraction profiles were obtained with the Circular Hough Transform plugin[1]. The diffraction lines of the β-Bi_2O_3 phase shown in Fig. 1f in the main manuscript were generated by means of VESTA software, starting from the crystallographic model for tetragonal β-Bi_2O_3 phase (space group P-421c). Regarding the sample preparation for TEM characterization, the catalyst powder was dispersed in pure ethanol and, after sonication, it was deposited on a lacey carbon Cu TEM grid by drop casting.

2. Electrochemical characterization

2.1. Electrode preparation

Typically, the synthesized catalyst (15 mg), acetylene carbon black (CB, Shawinigan Black AB50, 1.0 mg), Nafion® 117 solution (75 μL) and isopropanol (450 μL) was mixed by sonication for 40 min. Subsequently, the uniform slurry was drop-casted onto a carbon paper featuring a microporous layer (GDL; SIGRACET 28BC, SGL Technologies)[2,3]. The resulting GDE was left to dry at room temperature overnight. The catalyst loading is approximately 3.0 mg cm^{-2} and the active area of each GDE is 1.5 cm^2.

2.2. CO_2 electrolysis and product analysis

The electrolytes with different pH values were prepared by adjusting the acidity of 1.0 M KOH with concentrated H_2SO_4. The concentration of K^+ approximates to 1.0 M since the added volume of H_2SO_4 is very low. The CO_2RR experiments were conducted using the galvanostatic technique with a CHI760D potentiostat in a customized three-compartment three-electrode flow cell, as shown in Figure S1. When the acidic electrolyte was used, a proton exchange membrane (Nafion™ Membrane N117, Ion Power) was used to separate the anodic and cathodic sides, while an anion exchange membrane (Sustainion® 37-50, Dioxide materials) was employed with neutral and alkaline electrolytes. The cathodic side was divided by the GDE into catholyte and gas compartments. A miniature Ag/AgCl reference electrode (1 mm, specifically a leak-free LF-1 electrode) was employed and inserted into the catholyte. An Ir-coated Ti plate (Ir-MMO, 10 cm^2, ElectroCell Europe A/S) was

used as the counter electrode and immersed within the anolyte. In each measurement, the catholyte and anolyte are the same electrolyte, and were single-pass through the catholyte and anolyte compartments, respectively, at a flow rate of 4 mL min^{-1}. During the test, a constant CO_2 flow of 25 mL min^{-1} was maintained in the gas compartment to supply the reactant and to bring out the gaseous products.

For the real-time analysis of gas-phase products, a micro gas chromatograph (μGC, Fusion® model, INFICON) was employed. This μGC featured two distinct modules: one equipped with a 10-meter Rt-Molsieve 5A column with Ar as the carrier gas and the other with an 8-meter Rt-Q-Bond column with He as the carrier gas. Both modules were outfitted with micro thermal conductivity detectors (micro-TCD). Faradaic efficiency (FE), also known as current efficiency, serves as a crucial parameter in assessing the selectivity of an electrode towards a particular CO_2RR product. Equation 1 was applied to calculate the FE for a specific gaseous product.

$$\mathrm{FE} = \frac{V * t * C * n * F}{V_m * Q} \qquad \text{Equation 1}$$

where V_m is the molar volume of an ideal gas (L mol^{-1}); V is the gas flow rate at the cathodic side (L min^{-1}); t is electrolysis time (min); Q is the total charge passed through the system during the electrolysis time t (coulombs, C); C is the concentration of the gas product (% v/v); n is the number of electrons required to obtain 1 molecule of this product ($n = 2$ for CO and H_2 formation); F is the Faraday constant (96485 C mol^{-1}).

For the detection and quantification of liquid-phase products, a high-performance liquid chromatograph (HPLC, Thermo Scientific, Ultimate3000) was utilized. This HPLC system featured a UV-Vis detector set at a wavelength of 210 nm and a ReproGel chromatographic column with dimensions of 300 × 8 mm. The mobile phase used for the HPLC analysis was an aqueous solution of 9.0 mM H_2SO_4 delivered at a flow rate of 1.0 mL min^{-1}. The FE for each liquid product can be calculated from its concentration in the sampled catholyte using Equation 2.

$$\mathrm{FE} = \frac{v * C * n * F}{Q} \qquad \text{Equation 2}$$

where v is the volume of catholyte (L); C is the concentration of the liquid product (mol L^{-1}); n is the number of electrons required to obtain 1 molecule of this product ($n = 2$ for $HCOO^-$ formation); F is the Faraday constant (96485 C mol^{-1}); Q is the total charge passed through the system during the test (coulombs, C).

In the reported experimental data, the recorded electrode potentials were rescaled with respect to the Reversible Hydrogen Electrode (RHE) by applying the Nernst equation,

$$E_{RHE} = E_{Ag/AgCl} + E^0(Ag/AgCl) + 0.0591 * pH \qquad \text{Equation 3}$$

Where E_{RHE} is the potential with respect to the RHE; $E_{Ag/AgCl}$ is the applied potential with respect to the Ag/AgCl reference; $E^0(Ag/AgCl)$ is the standard potential of a Ag/AgCl (3 M Cl^-) reference with respect to standard hydrogen electrode (SHE); pH is the value of the bulk electrolyte.

3. *Operando* Raman Spectroscopy

Raman spectroscopic analyses were performed using a confocal Raman microscope (Renishaw InVia). Spectral data were collected with the Raman spectrometer coupled to a confocal microscope (Renishaw InVia) and operated by the WiRE5.3 software. The calibration was carried out using a silicon wafer standard (520.6 cm^{-1}). The excitation source was a 785 nm diode laser. The incident beam was attenuated to 5 % of initial power for measurements. An objective lens (50× magnification, 25 mm focal length, numerical aperture: 0.5) was applied for focusing and collecting the incident and scattered light. The acquisition time for each spectrum was 1.1 s, and 5 spectra, which were recorded at a given potential, were superposed to improve the signal-to-noise ratio. Raman spectra were collected in the range of 0 ~ 1120 cm^{-1}. For the data acquisition in the electrochemical experiments, we obtained a Raman spectrum every 5.5 s, converted into a given potential based on a scanning rate of 10 mV s^{-1}. A spectro-electrochemical cell (Ida, K008) with a three-electrode configuration and an optical window was used for electrochemical analyses. The counter electrode was a Pt mesh (10 mm × 10 mm). The reference electrode was a miniature Ag/AgCl electrode (saturated KCl solution) for acidic and neutral electrolytes, while a miniature Hg/HgO electrode (saturated KOH solution) was

used as the reference electrode in alkaline environments. Bi_2O_3-coated carbon paper was used as the working electrode. An electrochemical potentiostat (Bio-Logic SP-300) was used for controlling and measuring the potentials and currents. All reported potentials in this work were referred to the Reversible Hydrogen Electrode (RHE).

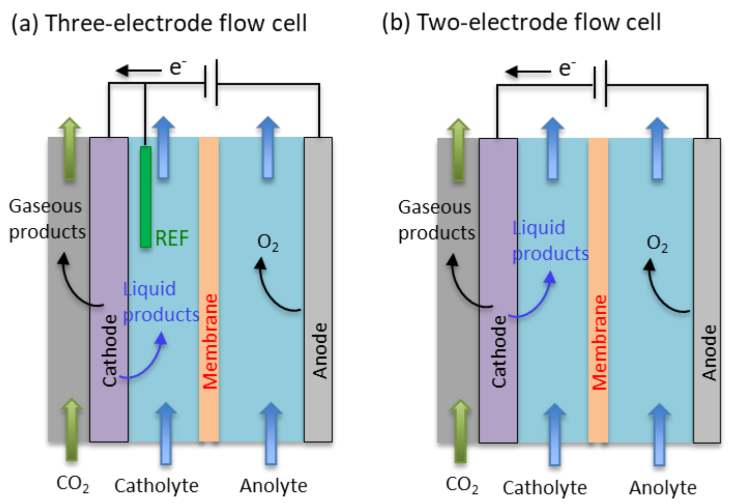

Figure S1. Schemes of the electrochemical cell (a) three-electrode and (b) two-electrode mode.

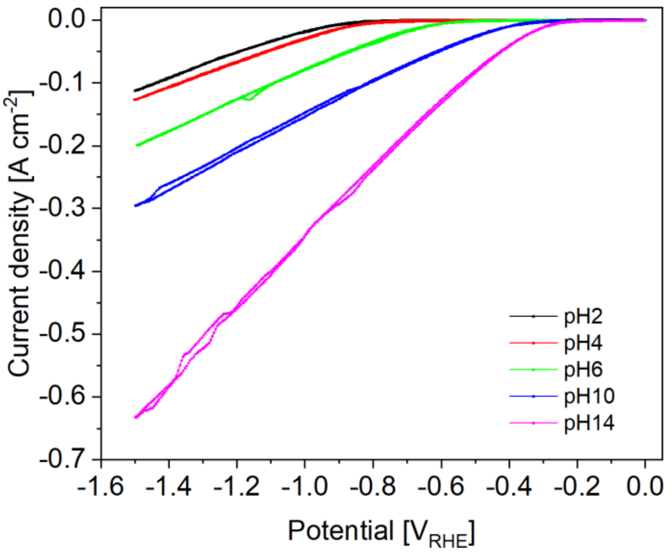

Figure S2. Linear sweeping voltammetry study on Bi-GDEs in electrolytes with different pH values in a flow cell.

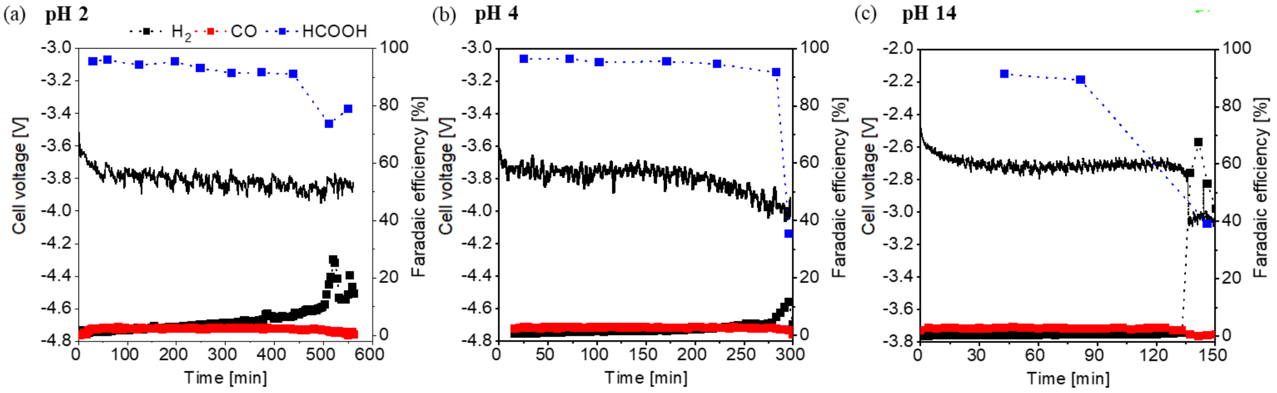

Figure S3. CO_2RR on Bi-GDEs with a two-electrode mode: product distribution at a current density of 400 mA cm^{-2} in different electrolytes (a) pH 2, (b) pH 4 and (c) pH 14.

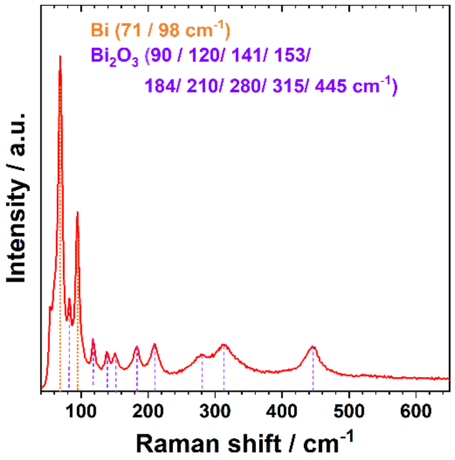

Figure S4. Raman spectrum of metallic Bi powder.

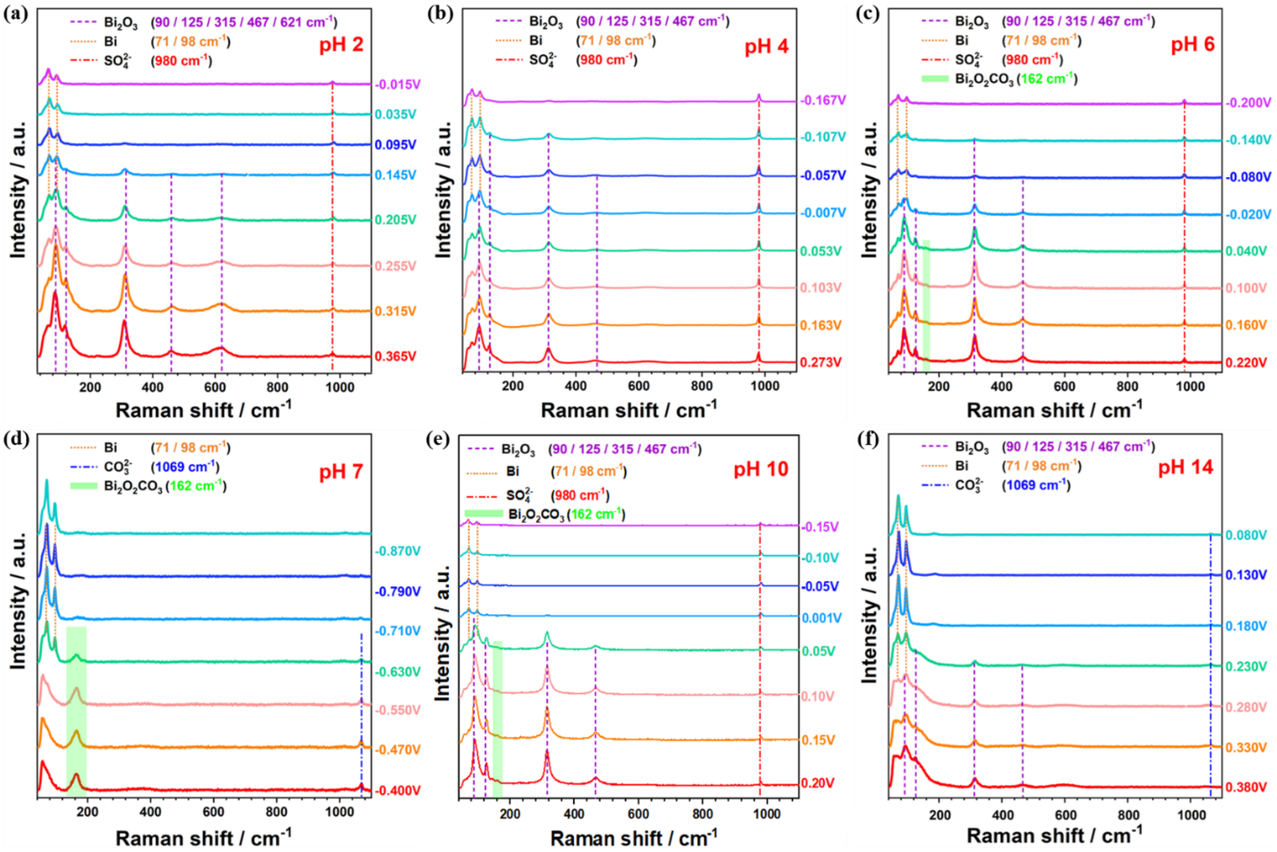

Figure S5. Operando electrochemical Raman spectra of Bi-GDEs in different electrolytes: (a) pH 2, (b) pH 4, (c) pH 6, (d) pH 7, (e) pH 10, and (f) pH 14.

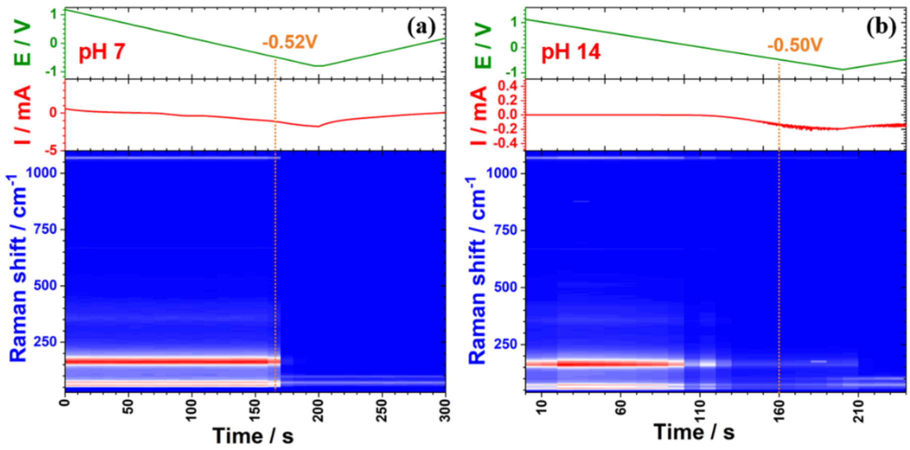

Figure S6. Raman intensity of Bi-GDEs prepared by $Bi_2O_2CO_3$ particles in (a) pH 7 and (b) pH 14 electrolyte.

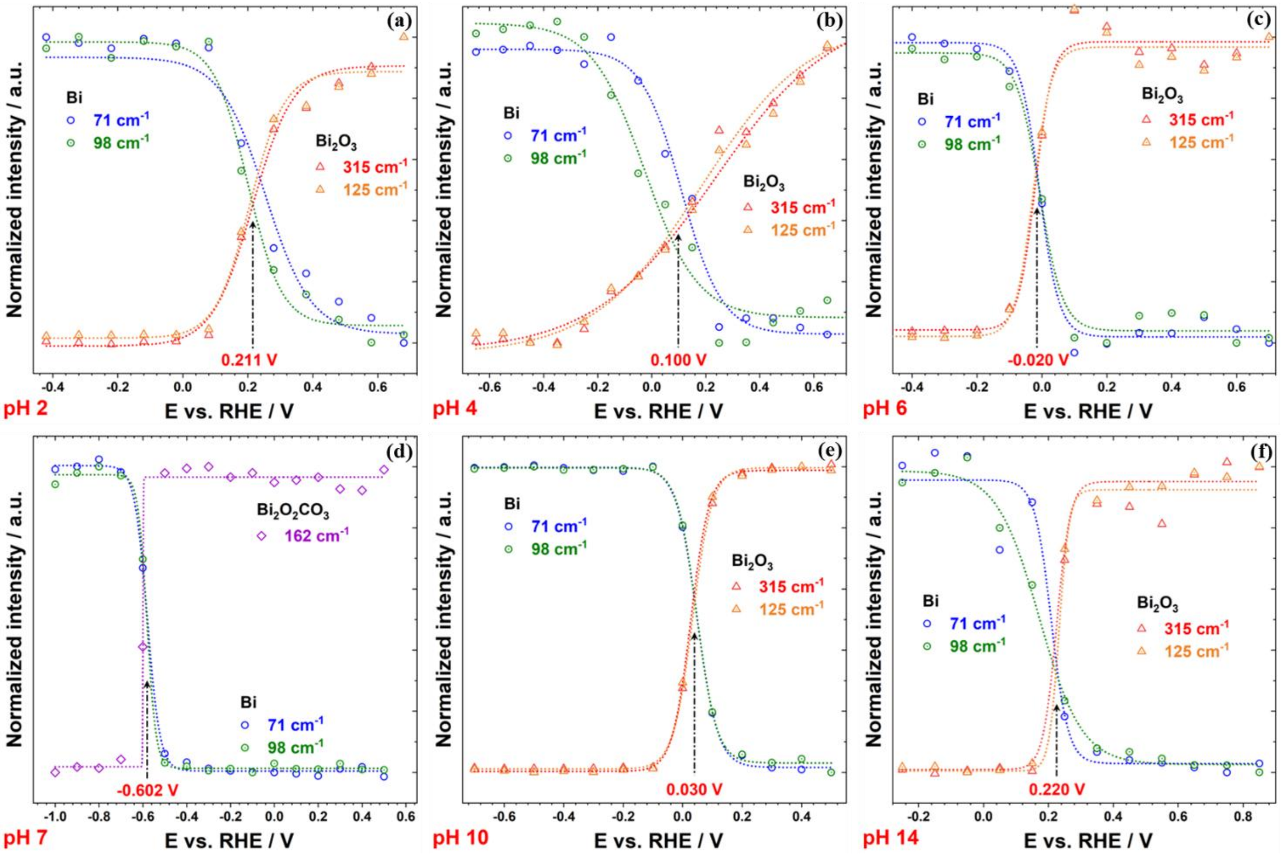

Figure S7. Boltzmann combination fitting results of the potential-dependent *operando* Raman spectra on Bi-GDEs in different electrolytes: (a) pH 2, (b) pH 4, (c) pH 6, (d) pH 7, (e) pH 10 and (f) pH 14.

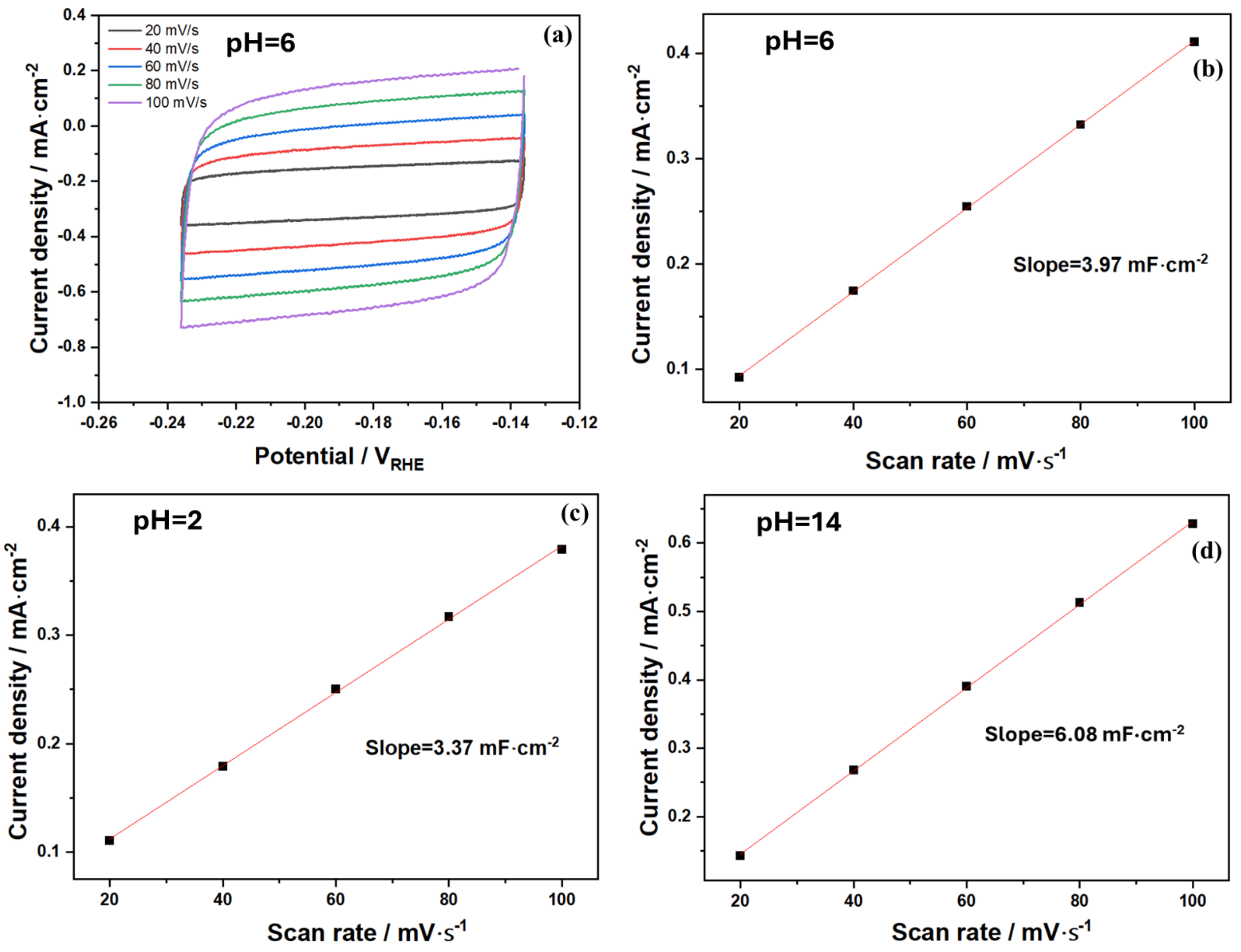

Figure S8. Evaluation of electrical double layer of Bi-GDEs in different electrolytes: (a) typical cyclic voltammograms at different scan rates in pH 6, (b) double layer capacitance (slope) in pH 6, (c) double layer capacitance (slope) in pH 2, (d) double layer capacitance (slope) in pH 14.

Reference

(1) Mitchell, D. R. G. Circular Hough Transform Diffraction Analysis: A Software Tool for Automated Measurement of Selected Area Electron Diffraction Patterns within Digital MicrographTM. *Ultramicroscopy* 2008, *108* (4), 367–374. https://doi.org/10.1016/j.ultramic.2007.06.003.

(2) Gavello, G.; Zeng, J.; Francia, C.; Icardi, U. A.; Graizzaro, A.; Specchia, S. Experimental Studies on Nafion® 112 Single PEM-FCs Exposed to Freezing Conditions. *Int J Hydrogen Energy* 2011, *36* (13), 8070–8081. https://doi.org/10.1016/j.ijhydene.2011.01.182.

(3) Zeng, J.; Nair, J. R.; Francia, C.; Bodoardo, S.; Penazzi, N. Aprotic Li–O_2 Cells: Gas Diffusion Layer (GDL) as Catalyst Free Cathode and Tetraglyme/$LiClO_4$ as Electrolyte. *Solid State Ion* 2014, *262*, 160–164. https://doi.org/10.1016/j.ssi.2013.09.032.
